# Southern Dental Association

**Published:** 1886-09

**Authors:** 


(TO BE CONTINUED.)

---

### SOUTHERN DENTAL ASSOCIATION.

EIGHTEENTH ANNUAL MEETING, HELD AT NASHVILLE, TENN., JULY 27, 28, 29 AND 30, 1886.

REPORTED FOR THE INDEPENDENT PRACTITIONER BY "MRS. M. W. J."

The Southern Dental Association held its eighteenth annual session in the spacious Lecture Hall of Watkin's Institute, Nashville, Tenn.

### OFFICERS.

*President*—W. C. Wardlaw, Augusta, Ga.
*First Vice-President*—B. H. Catching, Atlanta, Ga.
*Second Vice-President*—J. R. Knapp, New Orleans, La.
*Third Vice-President*—E. D. Hamner, Galveston, Tex.

*Corresponding Secretary*—E. S. Chisholm, Tuscaloosa, Ala.

*Recording Secretary*—R. A. Holliday, Atlanta, Ga.

*Treasurer*—H. A. Lowrance, Athens, Ga.

*Executive Committee*—W. H. Morgan, Nashville, Tenn.; G. F. S. Wright, Columbia, S. C.; W. H. Richards, Knoxville, Tenn.

FIRST DAY—FIRST SESSION.

The meeting was called to order by the President.

Dr. J. H. Prewitt, of Nashville, delivered an eloquent address of welcome, which was responded to by Dr. G. H. Winkler, of Augusta, Ga.

Dr. W. H. H. Thackston, the oldest living graduate in the profession, he having graduated in the second class from the Baltimore College, addressed the Association, delivering the message of the delegation from Virginia, a warm invitation to again visit the Old Dominion. He expressed his great gratification at meeting once more the friends of "auld lang syne," and also the young and rising representatives of the profession, and the corps of editors and journalists, to whom he accorded high meed of praise.

The President delivered his annual address, in which he took for his special topic the question: "Is dentistry a distinct profession, or should it be regarded as a specialty of medicine?" He took the ground that, whilst dentistry is a distinctly organized profession, made so by peculiar circumstances, it is properly and really a specialty of medicine, being at the same time a science as well as an art, and should be so recognized and encouraged. Though repudiated by the medical schools, it is as much a part of general surgery as ophthalmic, obstetric or laryngeal surgery. He discussed the subject thoroughly and logically, showing how impossible it was that one mind could ever properly cultivate the vast and ever-increasing domain of medicine, division of labor, whether mental or manual, also tending to proficiency of attainment. As we have the specialist of the eye, the ear, the thoracic cavity, the urinary organs, of nervous disorders, of skin diseases, of uterine affections, etc., why not the specialist of the mouth?

He spoke of the potent influence exerted by association gatherings in promoting the dignity of the profession, and of the natural fruits of these associations in laws regulating the practice of dentistry, in State Examining Boards, in the National Association of

Dental Examining Boards, and the National Association of Faculties of Dental Colleges, all of which exercise a reciprocal influence upon education, that foundation stone upon which the professional edifice must stand. The work should be pushed forward until a license granted in one State will be of binding force in all the States, and the diplomas of all the colleges received with respect and authority wherever presented. The culminating step should be the abolishing of all subordinate degrees, as D.D.S., D.M.D., L.D. S., etc., for a national degree, the equivalent in rank, reputation and dignity of the English degree, "Member of the Royal College of Surgeons." He set forth with pride the fact that we are even now leading the medical fraternity in the matters of State laws and professional education.

In the State of Georgia, at the very time when violations of the law against the illegal practice of dentistry were being successfully prosecuted, a committee of physicians was making futile efforts to prevent an oily-tongued, brass-mounted quack doctor from reaping a rich harvest from her deluded citizens. They were powerless to do more than grit their teeth and look on with smothered curses.

He suggested other matters for consideration and action, such as the appointment of dentists to the army and navy, and on the national board of health, our share in congressional appropriations for scientific research, the approaching medical congress, etc.

The address was listened to with the thoughtful attention it merited, and was followed by hearty applause.

The society then proceeded with the regular order of business.

The report of the Committee on Dental Education was called for.

Three papers were offered and read: One from Dr. B. H. Catching, one from Dr. W. D. Dunlap, and one from Dr. M. C. Marshall.

Dr. Catching considered the insufficiency of present modes of dental education; that while dentistry as an art had made wonderful progress, as a science it was advancing very slowly, and that separate dental schools were drawbacks to further advancement. We cannot expect to be recognized as medical specialists while we neither teach nor graduate as such.

It is commonly believed that to become a good dentist it is only necessary to have a mechanical turn of mind, and the only education necessary the training of such a bent. Separate schools have forced this conclusion. Before claiming recognition in medicine we must accept the teachings which qualify for it. In the dental schools very little is taught of theoretical, and nothing of clinical medicine.

Humanity demands that we should thoroughly qualify ourselves to heal her ills and remedy her defects.

To command the highest respect and attain the fullest sphere of usefulness we must ——

Do away with Dental Colleges;

Do away with University Departments;

Do away with the separate degree of D.D.S.;

We must enter reputable medical colleges for the regular degree of medicine. Then we should go to a dental infirmary where practical dentistry is taught by the best talent. This would make us fully equipped to practice dentistry as a specialty of medicine. Medical colleges should have chairs of dental surgery, and teach dentistry as applied to medicine. This would be of benefit to physicians and a blessing to the people, and cause a higher appreciation of educated dentistry.

Dr. W. D. Dunlap, in his paper (which was read by Dr. B. H. Teague), reviewed the disadvantages under which earlier students in dentistry labored, and applied the advantages of office training under a competent preceptor in combination with a college course. When the preceptor is competent, and does his duty, the student is daily at work carrying out the ideas of his preceptor. His hand is trained, his eye follows the work done by his master, and a firm foundation is laid. When he enters college he knows his deficiencies and his necessities. He is prepared to appreciate the instruction he receives. After a course of stuffing for five months he goes back to the office, where he has time to digest it, finally returning to be crammed again. When he receives his diploma and opens his office he has leisure to more fully digest the enormous meal furnished by the college. He deprecated the idea of graduating a man on the strength of what he knows, regardless of time. As it requires at least two years to make a blacksmith, or a carpenter, or a shoemaker, less should not be given the dentist for that thorough training of body and mind required to fit him for his life-work. He spoke of associations as the school of schools—the school of the prophets—where the hoary-headed professor tells of what he knows, the successful man how he succeeds, the timid how he fails. Every one is at liberty to criticise, to build up or pull down, to discriminate between the true and the false. In this great school no certificate of merit is given, no diploma conferred, no seal, no stamp on the brow with the inscription "I made you." Here there is no stamping as graduate a man who has only attended commencement exercises.

Education implies honest, earnest, continued labor for a purpose, with no shams, no slippings over. The signature of the educator should mean more than, "I have examined and found competent." It should mean, "I have materially aided in preparing him for his life-work."

Dr. M. C. Marshall considered the question of dental education from still another point of view, namely, the advantages of associated effort. He spoke of the chaotic condition of dentistry less than a quarter of a century ago, when everything pertaining to it was kept as a secret, to be disposed of for a consideration. He reviewed the history of the earlier organizations for associated effort, and the great results that have been accomplished through these means, the elimination of much that was narrow, bigoted and untenable, to be replaced by the spirit of progressive, philanthropic enlightenment, widening our capacity, cementing fraternal feeling; of the building up of a professional literature, which is the exponent of our attainments. State dental laws are another result of associated effort. In local societies our best thoughts are prepared for presentation at the State meetings, and thence to the wider field of the general Association.

He also spoke of our dental colleges, regretting that, under the present system, professorships were valuable in proportion to the number of matriculates, leading sometimes to the offer of too great inducements to students, or the guarantee of graduation in a specified time, for a stipulated fee. Without a preliminary examination, like the Procrustean bedstead, all were made to fit—the diploma ready if the fee is forthcoming. The very large number of colleges now in operation, and for which there is no demand, creates these unjustifiable practices. When the chairs are not profitable they will be filled by men of the same calibre as those in our State Legislatures, where honesty and ability rarely immolate themselves on their country's altar. To secure representative men in our college faculties they must be well compensated, endowment being probably the only plan.

These questions should engage the attention of the Southern Dental Association, which should seek to formulate a system of college matriculation and graduation that shall elevate the standard of dental education, and date an epoch in our professional existence.

DISCUSSION.

*Dr. J. J. R. Patrick*—Said that the subject was like the harp of a thousand strings. He deprecated the continual discussion of the question whether or not dentistry is a specialty of medicine, and gave a comprehensive retrospective view of the history of medicine and what the degree of M. D. is supposed to represent, concluding that now, as in the days of the aborigines, the "medicine man" should be the wisest man of the tribe. He paid a noble tribute to the memory of John Hunter, the first to describe the foetal circulation, the first to describe the human jaw, the human teeth, the father of the science of physiology.

*Dr. Wm. H. Morgan*—The statistician of dentistry, corrected some errors in dates mentioned in the history of early dental organization, and dental literature, and dental schools. He reviewed the hardships and trials of the first dental students, giving interesting reminiscences. He spoke strongly in favor of the present system of dental college education, deprecating the views expressed in the papers read.

*Dr. B. H. Catching*—Upheld the views expressed in his paper, as the result of strong conviction and mature consideration. He considered that, as a profession, we cannot longer occupy our present position. We must either go backward or forward, professional pride demanding that it be forward, and into the domain of medicine. He considered that the dental student of the future should first graduate an M. D. and then enter a dental infirmary to acquire the practical details and the manipulative art.

*Dr. O. Salomon*—Thought that Dr. Catching had portrayed the ideal future of dentistry, but that it was impracticable at present. He considered that either dental schools must extend their course of medical study, or medical schools must enlarge their dental facilities. He would not cast reflections upon his Alma Mater, but urge the old colleges to adopt higher standards.

*Dr. B. H. Teague*—Strove to reconcile the opposing views of men who spoke from different standpoints, and discussed the subject from both points of view. He considered that the place to make the medical man was the medical college, but the place for the dental student was the dental college. He deplored the disrespect brought upon American dentistry abroad by the action of disreputable colleges in sending out incompetent men. He said that the present dental college system requires overhauling. Students must be better educated. There must be a higher standard of admission, and the preceptor also must be known to be properly qualified.

*Dr. McKellops*—Spoke from the standpoint of a self-made man, congratulating the students of to-day, in most forcible language, on the grand opportunities they enjoy, but which they so carelessly neglect or recklessly throw away. He compared the days when everything connected with a dental office was held strictly secret, to be paid for, even if it were only how to make soft solder, with the cordial fraternal reception accorded professional brethren to-day. He himself put the latch-string out, and said to every man: "You are welcome to my office and to learn all you can in it." His patients never refused to allow one of his brother dentists to stand by his chair and study his operations. He spoke of the change taking place in the reception accorded to dentists by the medical profession, many being members of both professions, the favorites of one and the pride of the other. He spoke of the methods of Herbst, with whom he had just spent ten days in New York, and who says: "I have brought my baby to this country; I want to show it to you. If you don't like it, I'll drown it."

*Dr. W. H. H. Thackston*—Said that in the earlier efforts towards dental education, alliance was sought with medical colleges, but the overtures were rejected; that we had a right to be proud of the results of a separate, independent system of dental education, and that it should not be hastily abrogated and abandoned simply because some men under that system were not what they ought to be; that no system in the world could force ideas through thick skulls, or fill empty heads with brains; that there were quacks in all professions, and noodle-heads everywhere,—in medicine, in law, in the ministry. He considered that it would be making too great concessions of dignity and of self-respect to go back now to the medical schools which so long gave us the cold shoulder. There were very few men who were fit to be dental cadets, though they might make respectable physicians or lawyers. When the millennium comes it will make very little difference whether we attend medical or dental schools, but until that time we should be very careful not to make any serious departures from established practices.

*Dr. G. F. S. Wright*—Spoke of the influence exerted by State associations in fostering dental education and enacting laws for the regulation of practice; but with all that could be done, men already in practice could not be interfered with, no matter what their defects. It is a principle in common law that the law cannot make a crime of what was not a crime before the law was enacted, but that the time was coming when every State would have a good and effective law, under which it would not be possible for a man to enter the profession unless he were really qualified.

*Dr. Jas. Johnson*—Spoke to the same point, that a law had been passed in Virginia which they hoped to make effectual. A great many are now in practice whom we cannot reach. We shall have to be patient till they die off, for we cannot touch them. He also thought, after looking the matter over carefully, that what we need is the establishment of universities with endowments. With well-endowed chairs we could get the best that the profession has. As things now are, we have not the means to pay for the best talent.

*Dr. Crawford*—Said that in a large per cent of the literature of dentistry, fundamental principles are viewed from a medical standpoint. In our periodical literature the best articles come from men who sign M. D., with perhaps the addition of D.D.S., but the M. D. takes precedence. The idea that the dentist must be a doctor has come to stay. The man who has had the training and education necessary to secure the degree of M. D. comes to the front.

*Dr. D. R. Stubblefield*—Spoke from the college standpoint. He said that if we could have dental students who were graduates from a literary college, or even from a high school, we would have a broad foundation on which to put the capstone. But this not being the case, we must do the best we can with the material that comes to us. He said that he himself was educated for the medical profession, but had left it for dentistry because God had given him mechanical instincts which find their proper outlet in dentistry.

That the school, whether medical or dental, made but little difference in the end.

*Dr. Morgan*—Said that previous speakers made a mistake in supposing the possession of certain attainments entitled a man to a degree. This was not so. A degree was conferred by certain institutions on certain conditions, and unless all those conditions were fulfilled there was no ground for claiming the degree. Not all the knowledge of an archangel could *entitle* a man to a degree until he had complied with the conditions of the institution conferring it.

*Dr. McKellops*—Asked whether a man was not entitled to a diploma if he presented himself for examination and passed it honorably?

*Dr. Morgan*—No, sir; not unless he complies with all the conditions with regard to fees, curriculum, etc. The percentage of thoroughly educated men in the two professions, in proportion to the number of men employed in each, is largely in favor of dentistry—ten to one, in fact. He considered the average office training a disadvantage and detrimental. He would rather take a young man from the plough than from the average dental office. He thought, also, that much that was taught in medical schools was of no practical use to the dentist, as, for instance, the treatment of yellow fever or cholera.

*Dr. Wright*—Asked about the conditions on which honorary degrees were conferred.

*Dr. Morgan*—Said that they were only bestowed upon men who had made valuable contributions to literature or science ; only when well-merited, and where it was certain they could do no harm to the profession.

*Dr. R. B. Adair*—Thought the standard of literary acquirements now required by college faculties a proof of real advancement.

Dr. Morgan introduced Dr. G. W. Hubbard, the Dean of Meharry Medical College, an institution for the professional education of colored people, which will open, in October next, a dental department.

Dr. Hubbard spoke at some length in favor of the institution, showing what had been accomplished in the medical department, which has 62 medical graduates now in practice, the three years' course being very thorough and the standard of graduation high, with very strict examination. The dental course will be of two terms, extended to three where found necessary, and he bespoke for their dental graduates the same welcome from the dental profession that the medical profession had granted to their medical graduates. He hoped they would also make it known among intelligent colored men. The work is extremely philanthropic, the terms being $30.00 a session; $75.00 or $80.00 covering all expenses of the course, including materials, graduation fee, etc. There is also a literary department, where those who are not able to pass the preliminary examination will be entered for a year. The only requirement is a good moral character, soberness and uprightness, without regard to color.

On motion, the subject of Dental Education was passed.

